# Process evaluation of a systemic intervention to identify and support partner violence survivors in a multi-specialty health system

**DOI:** 10.1186/s12913-020-05809-y

**Published:** 2020-10-31

**Authors:** Emma C. Jackson, Lynette M. Renner, Nyla I. Flowers, Mary E. Logeais, Cari Jo Clark

**Affiliations:** 1 Hubert Department of Global Health, Rollins School of Public Health, Emory University, Atlanta, Georgia USA; 2 School of Social Work, University of Minnesota, Minneapolis, Minnesota USA; 3 Medical School, University of Minnesota, Minneapolis, Minnesota USA

**Keywords:** Intimate partner violence, Screening, Referral, Facilitators, Barriers, Consolidated framework for implementation research

## Abstract

**Background:**

Intimate partner violence (IPV) is highly prevalent in the United States and impacts the physical and mental health and social well-being of those who experience it. Healthcare settings are important intervention points for IPV screening and referral, yet there is a wide range of implementation of IPV protocols in healthcare settings in the U.S., and the evidence of the usefulness of IPV screening is mixed. This process evaluation investigates the facilitators and barriers to implementing *Coordinated Care for IPV Survivors through the M Health Community Network (“M Health Network”),* an intervention that aimed to standardize IPV screening and referral in a multi-specialty clinic and surgery center (CSC). Two validated IPV screens were introduced and mandated to be done by rooming staff at least once every 3 months with all clinic patients regardless of gender; the Humiliation Afraid Rape Kick (HARK) for presence of IPV and the shortened Danger Assessment (DA-5) for lethality of IPV. Upon a positive screen, the patient was offered immediate informational resources and, if willing, was referred to a social worker for care coordination with a community organization.

**Methods:**

Semi-structured, individual and group process interviews with clinic managers and clinic staff at 8 CSC clinics (*N* = 24) were undertaken at 3,12, and 27 months after intervention start. Semi-structured interviews were undertaken with the research team (*N* = 3) post-implementation. A Consolidated Framework for Implementation Research (CFIR) codebook was used to code data in two rounds. After each round, thick description was used to write detailed and contextual descriptions of each code. Facilitators and barriers to implementation were identified during the second round of thick description.

**Results:**

Facilitators to implementation were clinic staff support, dedication, and flexibility and research team engagement. Barriers were lack of prioritization, loss of intervention champions, lack of knowledge about intervention protocol and resources, staff and patient discomfort discussing IPV, and operational issues with screen technology.

**Conclusions:**

The IPV protocol was implemented, but faced common barriers. CFIR is a complex, but comprehensive, tool to guide process evaluation for IPV screening and referral interventions in health systems in the U.S.

## Background

Intimate partner violence (IPV), or the perpetration of any psychological, physical, or sexual violence by a current or former intimate partner, is a serious public health problem [1]. IPV leads to severe physical, mental, and social health impacts for those who experience it, including death [2–5]. Female survivors of IPV in the United States have increased annual healthcare utilization that can continue for years after exposure to violence ends [6, 7]. Congruently, healthcare professionals are often the first professionals to speak with people about their experiences of IPV [8, 9]. For these two reasons, healthcare services are a critical intervention point for IPV screening and referral.

The National Academy of Medicine (formerly the Institute of Medicine) and the U.S. Preventive Services Task Force (USPSTF) recommend that women of childbearing age be screened for IPV by clinicians, citing evidence for minimal risk for harm [10]. Although there is evidence that IPV screening in a clinical setting is not harmful to women in the short-term, [11] the evidence for the usefulness of screening is mixed. USPSTF’s 2018 recommendation concludes that IPV screens can accurately detect IPV in women, but that there is insufficient evidence for screens detecting IPV in men, [12–16] that there is insufficient evidence that screening reduces IPV, and that there is insufficient evidence that screening is non-harmful for men [10]. Non-binary gender identities are not addressed in this recommendation and most existing tools were not designed to measure IPV among identities other than “woman” [17]. A 2012 systematic review and meta-analysis of studies to assess the effectiveness of IPV screening in healthcare settings found that screening likely increases identification of IPV, but found no evidence for improved health or harm outcomes for women screened for IPV [11]. This same study highlighted that even though screening may increase identification of IPV, the rates of identification are low compared to the known prevalence of violence [11]. In terms of referral, the same study found that across trials, introducing screening did not necessarily lead to increased effective referral for patients [11].

The mixed evidence on the usefulness of IPV screening may be due to a lack of clear, evidence-based guidelines for best-practices for implementing IPV screening. There are several identified facilitators and barriers to implementing IPV screening. Screening protocols that use validated screens consistently and include a pre-formulated script may have more success in identifying IPV [18, 19]. Other factors that contribute to successful IPV screening are consistent and repetitive training of healthcare providers and immediate care coordination (i.e., referring patients to resources for IPV) for patients who screen positive [19]. There are several well-documented barriers to effective IPV screening in healthcare settings that span medical specialty [20–22]. These include lack of time and limited incentive for providers to perform IPV screening, lack of policy to guide providers in screening, discomfort on the part of providers and patients in discussion of violence, lack of training, and lack of healthcare collaboration with community organizations that provide IPV care [20, 21, 23]. For patients, fear of further violence from the perpetrator or fear of children being taken away may serve as barriers to disclosure [24].

USPSTF also recommends that healthcare settings systematize referral to ongoing support services for women who screen positive for IPV based on evidence that IPV interventions that include this can reduce violence, abuse, and physical or mental health impacts for pregnant and post-partum women [10, 24–26]. This evidence, along with a 2016 review of literature from 2000 to 2015 that found that those who screened positive for IPV in emergency room settings were more likely to experience abuse in the months after detection, highlights the importance of including timely referral to ongoing support in addition to IPV screening in healthcare settings [27]. Despite the recommendation and evidence, a 2017 review of 35 U.S. studies published at any time on IPV screening and counseling revealed that a range of 2–50% of medical providers report consistent IPV screening for women [28].

### Study purpose

We evaluated the process of implementing a unique, systemic IPV screening and referral protocol intervention at a multi-specialty clinics and surgery center in the Midwestern United States. The evaluation was designed using the Consolidated Framework for Implementation Sciences (CFIR) [29]. The CFIR is a tool developed by implementation researchers to identify potential barriers or facilitators to intervention implementation and to aid in systematic evaluation of implementation contexts [30]. This qualitative analysis aims to utilize the five domains of implementation laid out in the CFIR; Intervention Characteristics (*key attributes of interventions that influence the success of implementation*), Outer Setting (*characteristics of and relationships with external entities related to implementation*), Inner Setting (*characteristics of the implementing organization that influence the success of implementation*), Characteristics of Individuals (*characteristics of individuals involved in implementation*), and Process (*how implementation is enacted*) to evaluate the barriers and facilitators to the implementation of the IPV protocol.

### Intervention description

In 2014, researchers at a University in the Midwest conducted a pilot study to better understand the protocol and practices related to IPV screening and referral at one academic primary health clinic. In response to findings from the pilot study, [31] the *Coordinated Care for IPV Survivors through the M Health Community Network (“M Health Network”)* intervention was implemented to expand and standardize the IPV screening and referral system across a multi-specialty clinic and surgery center (CSC), and to operationalize and standardize documentation and communication across healthcare providers and a network of community organizations. This activity together comprised the implementation of a novel IPV protocol at the CSC. The intervention was a collaboration between the research team from the university, the CSC, and a community-based organization that provided legal counsel, therapy, and case management to survivors of IPV. The CSC has 37 adult medical specialty clinics that served 2000–2500 patients daily. This sub-specialty driven practice consisted of approximately 1000 physicians, of which 10–15% were in primary care. Patients accessing healthcare services at the clinics mostly resided in the metropolitan area of the state capital, but referrals came from across the state.

The novel IPV protocol stipulated that all adult patients, regardless of sex or gender identity, should be screened for IPV by clinic rooming staff (licensed practical nurses, medical assistants, and emergency medical technicians) at least every 3 months using an EPIC™ screening tool. The IPV screen was based on a validated, USPSTF-endorsed, tool called the HARK (Humiliation, Afraid, Rape, Kick) screen, [16] and on a validated, five-item version of the Danger Assessment (DA-5) [32]. The HARK was used to screen for the presence of IPV and the DA-5 was used to assess for lethality among patients who screened positive for IPV. If a positive result occurred on the HARK, the DA-5 was completed. A score of 2 out of 5 on the DA-5 was considered highly predictive of risk for serious assault or homicide. If a patient disclosed recent IPV, they were asked whether they would like to receive any referrals, including printed information on community resources or speaking with a member of the Behavioral Health Team (typically a licensed clinical social worker).

**Fig. 1:**
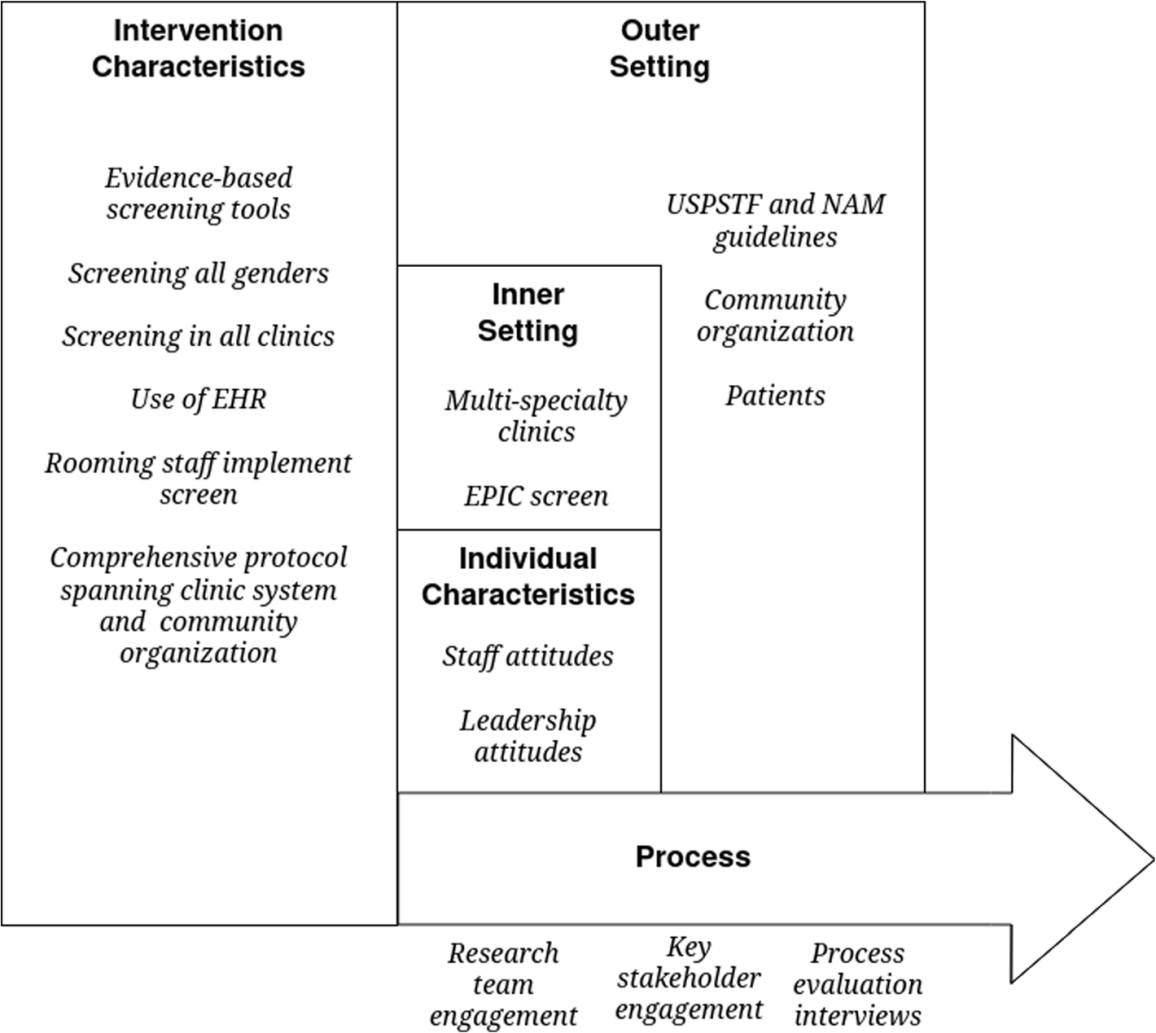
CFIR framework with examples from M Health Network IPV Protocol intervention

If the patient opted to speak with a Behavioral Health Team member, that provider conducted an in-clinic assessment of patient safety and mental health and made connections to applicable legal, financial, mental health, and housing resources. Depending on the priorities identified, the patient could be connected to the case manager at the community organization. The case manager carried a dedicated cell phone to speak with these patients. If the patient preferred to contact the case manager independently, they were offered a nondescript business card with the agency’s telephone number and a crisis hotline, and/or a list of IPV-focused community resources, with effort taken to discuss the safety implications of written materials with the patient. The case manager was also available to meet directly with patients at the clinics at the time of the positive screen. This process in entirety was considered “care coordination”. Use of the IPV protocol at the CSC commenced on November 15th, 2016. Questions and issues with the new screen were fielded immediately by the research team. Cardiology, represented in this analysis, did not have access to the IPV screen in EPIC™ until February, 2017, due to technical difficulties. All aspects of the *M Health Network* intervention can be mapped to the CFIR framework (Fig. 1).

Intervention activities included: 1) engaging hospital and community stakeholders, 2) developing the novel IPV protocol, 3) creating the IPV electronic health record screening tool using EPIC™ software, and 4) training CSC staff to implement the screen and response protocol. Training was online and focused on the new IPV screening and response protocol in the CSC. The training included definitions and basic facts related to IPV and its association with health; how to ask the evidence-based screening items and how to respond to disclosures of IPV; and how to connect patients to the appropriate in-clinic staff who would then conduct a broader assessment and connect patients to community services. The online training was required for all existing CSC staff who had patient contact, except for physicians, and remained open for new employees. In addition, 19 social work and behavioral health staff participated in an introductory, in-person training on the new IPV protocol in the Fall of 2016. Between the Fall of 2016 and the Summer of 2019, a total of 1694 CSC employees completed the online training. Prior to the IPV screen going live and shortly thereafter, nine in-person trainings and one Web-Ex training were held on the new IPV screen and response protocol, largely due to request from the clinics. These trainings were open to all clinic staff but were largely attended by rooming staff who conducted the screen. A total of 135 staff participated in supplemental trainings.

Originally, the IPV screen was intended to be implemented in the 13 clinics at the CSC with the highest female patient populations. These clinics were: cardiology, dermatology, endocrinology, neurology, oncology (four separate clinics), orthopedics and sports medicine, otolaryngology (i.e., ear, nose, and throat), primary care, pulmonology, and rheumatology. As the research team worked with the EPIC™ builder to include the IPV screen in the electronic health record, they discovered that all 37 clinics in the CSC (and some outside the CSC, such as psychiatry) would have access to the IPV screen. Because of this, the focus of the intervention expanded from the 13 selected clinics to all the clinics that had access to the IPV screen through the CSC’s electronic health record.

## Methods

### Data collection

Data for this analysis come from semi-structured individual and group process interviews with clinic managers, rooming staff, and social workers at the 13 clinics initially selected to implement the IPV screen and at the psychiatry clinic, and the CSC executive director (*N* = 24). Process interviews were undertaken at 3, 12, and 27 months after the screen went live (Table 1). At each intervention time-point, the research team contacted clinic managers via email to schedule process interviews with them and with the clinic’s rooming staff and social workers. Clinic managers who responded were asked to schedule a date and time based on the availability of their staff. Process interviews with rooming staff and social workers were open to all who could attend, and often had more than one staff member present (group interview size ranged from 2 to 12 participants). Clinic managers were interviewed individually. Clinics represented in the data include primary care, cardiology, psychiatry, oncology, otolaryngology, orthopedics and sports medicine, rheumatology, and dermatology. Clinics not represented in the data did not respond to the research team’s requests to be interviewed. Saturation was not the goal of selecting interviewees. Rather, the goal was to hear from as many clinic managers and staff across the 13 clinics (plus psychiatry) as possible at each time-point during implementation. To augment process data, informal, semi-structured interviews were conducted with research team members post-implementation of the IPV protocol (*N* = 3). The research team was interviewed in their roles as intervention designers and qualitative data collection facilitators. IRB approval for the project was granted by the University of Minnesota (1512 M80888) and Emory University (IRB00094148).

**Table 1:** Semi-structured process interviews with CSC clinic staff and leadership

	Follow-up 1 (3 months)	Follow-Up 2 (12 months)	Follow-Up 3 (27 months)
**Clinic**	January–February 2017	November–December 2017	February 2019
Cardiology^a^		Clinic Manager, Rooming Staff	Clinic Manager, Lead Rooming Staff
Dermatology	Clinic Manager, Rooming Staff	Clinic Manager	
Endocrinology			
Neurology			
Oncology	Rooming Staff	Clinic Manager, Rooming Staff	Clinic Manager, Rooming Staff
Orthopedics & Sports Medicine		Clinic Manager, Rooming Staff	Rooming Staff
Otolaryngology	Clinic Manager, Rooming Staff	Clinic Manager, Rooming Staff	Clinic Manager, Rooming Staff
Primary Care		Supervisor, Floor Manager, Senior Rooming Staff	Clinic Manager
Psychiatry^b^		Clinic Manager, Rooming Staff, Clinic Social Worker	Clinic Manager, Rooming Staff
Pulmonology			
Rheumatology		Clinic Manager	
CSC Leadership		CSC Executive Director	

The IPV screen went live in November 2016. Follow-up interviews were held approximately 3, 12, and 27 months later

^a^Four separate cardiology clinics are included in interviews

^b^Psychiatry is not part of the CSC; however, changes to EPIC affected other University Clinics. Psychiatry staff work with many patients who experience abuse and wanted to be active users of the screen. Thus, this clinic was included in data collection efforts

The individual and group process interviews were conducted on-site by one of the two Principal Investigators of the research team, audio recorded, and then transcribed at a later date. The research project manager manually transcribed all the interviews collected at 3 and 12 months, while interviews collected at 27 months were transcribed by a company called Verbal Ink. Permission to record was obtained from all participants in the study prior to commencement of the interview. Each interview lasted approximately 30 min. The researcher who conducted the interviews is a professor of social work, holds a clinical social work license, and has extensive training in qualitative methods. The researcher had a prior relationship with some of the CSC leadership, and some clinic managers and rooming staff at the primary care clinic. Other individuals in attendance during some of the interviews included the research project manager and a doctoral-level graduate research assistant. The interview questions were devised for the project. The midpoint and endpoint tools were based explicitly on CFIR domains and constructs and covered topics such as: the general level of receptivity to implementing the IPV screen and response protocol; how well the revised screen and response protocol fit with existing clinic practices; the priority of screening and responding to IPV relative to other initiatives that were happening in each clinic; what positive interactions with patients resulted from implementation of the IPV screen and response; how the screening and response protocol could be improved to better meet patient needs; what endorsement or support from clinic leadership had been seen or heard, what were overall successes and challenges to screening; and what further support was needed for staff. Copies of the interview questions were provided to clinic managers (See *Process Evaluation Questions*).

*S*emi-structured interviews with the research team post-implementation were undertaken remotely and in-person in February and March of 2019 by a masters-level graduate research assistant trained in qualitative methods who had a working relationship with one of the team members. The graduate research assistant transcribed notes while the interviewees answered questions. These interviews were audio recorded with permission from the interviewees. Questions in this interview guide focused on CFIR domains and constructs not present in the process data, particularly around the constructs of intervention source and complexity under the intervention characteristics domain.

### Analysis

The codebook was created prior to analyzing the data using the five domains and associated constructs of each domain laid out in the CFIR framework (Table 2). The five domains used as key codes were: 1) Intervention Characteristics, 2) Outer Setting, 3) Inner Setting, 4) Characteristics of Individuals, and 5) Process. During the first round of coding, two graduate research assistants coded transcribed interviews using these five key codes. During the second round of coding, the same graduate research assistants coded the data coded into these 5 key codes using the CFIR constructs (i.e., sub-codes) for each of the five domains. For example, Intervention Characteristics has the following associated constructs: *adaptability, complexity, cost, design quality and packaging, evidence strength and quality, intervention source, relative advantage, and trialability.* After the first round of coding, all interview text coded as “Intervention Characteristics” was compiled and re-coded into the associated constructs. This was done for all five CFIR codes. An inter-coder reliability of 67% was achieved between the two coders along with training to standardize understanding of the CFIR codes prior to initiating formal coding. All coding was done using MaxQDA™.

**Table 2:** CFIR framework codebook

**Domain 1: Intervention Characteristics**	
Intervention Source	Perception of key stakeholders about whether the intervention is externally or internally developed.
Evidence Strength and Quality	Stakeholders’ perceptions of the quality and validity of evidence supporting the belief that the intervention will have desired outcomes.
Relative Advantage	Stakeholders’ perception of the advantage of implementing the intervention versus an alternative solution.
Adaptability	The degree to which an intervention can be adapted, tailored, refined, or reinvented to meet local needs.
Trialability	The ability to test the intervention on a small scale in the organization, and to be able to reverse course (undo implementation) if warranted.
Complexity	Perceived difficulty of the intervention, reflected by duration, scope, radicalness, disruptiveness, centrality, and intricacy and number of steps required to implement.
Design Quality and Packaging	Perceived excellence in how the intervention is bundled, presented, and assembled.
Cost	Costs of the intervention and costs associated with implementing the intervention including investment, supply, and opportunity costs.
**Domain 2: Outer Setting**	
Patient Needs and Resources	The extent to which patient needs, as well as barriers and facilitators to meet those needs, are accurately known and prioritized by the organization.
Cosmopolitanism	The degree to which an organization is networked with other external organizations.
Peer Pressure	Mimetic or competitive pressure to implement an intervention; typically because most or other key peer or competing organizations have already implemented or are in a bid for a competitive edge.
External Policies and Incentives	A broad construct that includes external strategies to spread interventions, including policy and regulations (governmental or other central entity), external mandates, recommendations and guidelines, pay-for-performance, collaboratives, and public or benchmark reporting.
**Domain 3: Inner Setting**	
Structural Characteristics	The social architecture, age, maturity, and size of an organization.
Networks and Communications	The nature and quality of webs of social networks and the nature and quality of formal and informal communications within an organization.
Culture	Norms, values, and basic assumptions of a given organization.
Implementation Climate	The absorptive capacity for change, shared receptivity of involved individuals to an intervention, and the extent to which use of that intervention will be rewarded, supported, and expected within their organization.
Tension for Change	The degree to which stakeholders perceive the current situation as intolerable or needing change.
Compatibility	The degree of tangible fit between meaning and values attached to the intervention by involved individuals, how those align with individuals’ own norms, values, and perceived risks and needs, and how the intervention fits with existing workflows and systems.
Relative Priority	Individuals’ shared perception of the importance of the implementation within the organization.
Organizational Incentives and Rewards	Extrinsic incentives such as goal-sharing awards, performance reviews, promotions, and raises in salary, and less tangible incentives such as increased stature or respect.
Goals and Feedback	The degree to which goals are clearly communicated, acted upon, and fed back to staff, and alignment of that feedback with goals.
Learning Climate	A climate in which: a) leaders express their own fallibility and need for team members’ assistance and input; b) team members feel that they are essential, valued, and knowledgeable partners in the change process; c) individuals feel psychologically safe to try new methods; and d) there is sufficient time and space for reflective thinking and evaluation.
Readiness for Implementation	Tangible and immediate indicators of organizational commitment to its decision to implement an intervention.
Leadership Engagement	Commitment, involvement, and accountability of leaders and managers with the implementation.
Available Resources	The level of resources dedicated for implementation and on-going operations, including money, training, education, physical space, and time.
Access to Knowledge and Information	Ease of access to digestible information and knowledge about the intervention and how to incorporate it into work tasks.
**Domain 4: Characteristics of Individuals**	
Knowledge and Beliefs about the Intervention	Individuals’ attitudes toward and value placed on the intervention as well as familiarity with facts, truths, and principles related to the intervention.
Self-efficacy	Individual belief in their own capabilities to execute courses of action to achieve implementation goals.
Individual Stage of Change	Characterization of the phase an individual is in, as he or she progresses toward skilled, enthusiastic, and sustained use of the intervention.
Individual Identification with Organization	A broad construct related to how individuals perceive the organization, and their relationship and degree of commitment with that organization.
Other Personal Attributes	A broad construct to include other personal traits such as tolerance of ambiguity, intellectual ability, motivation, values, competence, capacity, and learning style.
**Domain 5: Process**	
Planning	The degree to which a scheme or method of behavior and tasks for implementing an intervention are developed in advance, and the quality of those schemes or methods.
Engaging	Attracting and involving appropriate individuals in the implementation and use of the intervention through a combined strategy of social marketing, education, role modeling, training, and other similar activities.
Opinion Leaders	Individuals in an organization who have formal or informal influence on the attitudes and beliefs of their colleagues with respect to implementing the intervention.
Champions	Individuals who dedicate themselves to supporting, marketing, and ‘driving through’ an [implementation], overcoming indifference or resistance that the intervention may provoke in an organization.
External Change Agents	Individuals who are affiliated with an outside entity who formally influence or facilitate intervention decisions in a desirable direction.
Executing	Carrying out or accomplishing the implementation according to plan.
Reflecting and Evaluating	Quantitative and qualitative feedback about the progress and quality of implementation accompanied with regular personal and team debriefing about progress and experience.

Adapted from CFIR website: https://cfirguide.org/constructs/

After each round of coding, data were analyzed using thick text description [33]. The use of thick description allowed for depth and nuance in the key domains and related constructs to be explored. The second round of thick descriptions provided a chance to review and embellish the thick description created after the first round of coding. Due to the inter-relatedness and repetition of findings across CFIR domains, and the aim of implementation science to generate information about facilitators and barriers to implementation, [34] we conducted the second round of thick descriptions focusing on facilitators and barriers. Results are organized below within the two broader categories. Saturation of data was achieved, evidenced by the repetition of themes across the five CFIR domains.

## Results

**Fig. 2:**
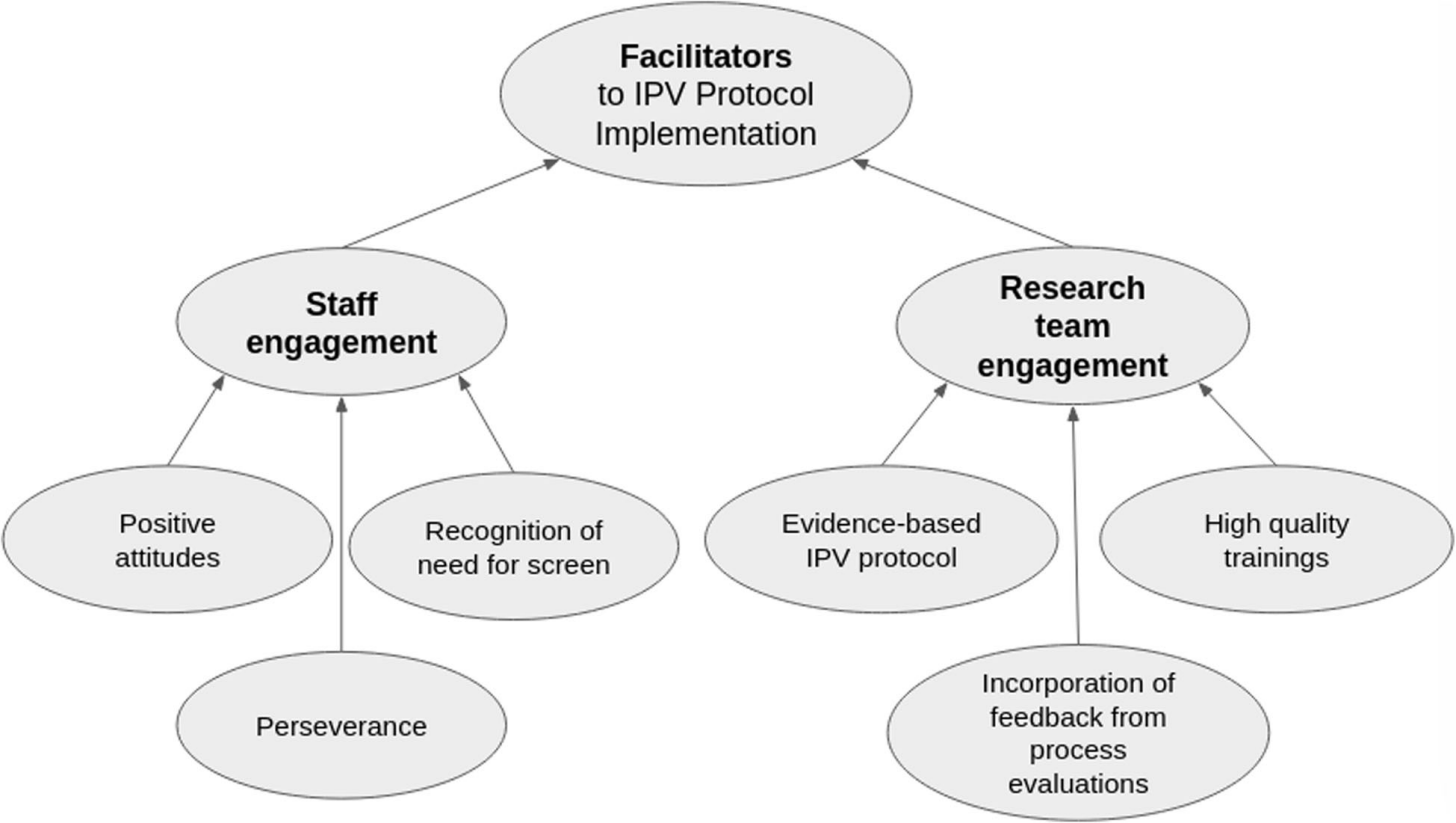
Facilitators to IPV Protocol implementation

**Fig. 3:**
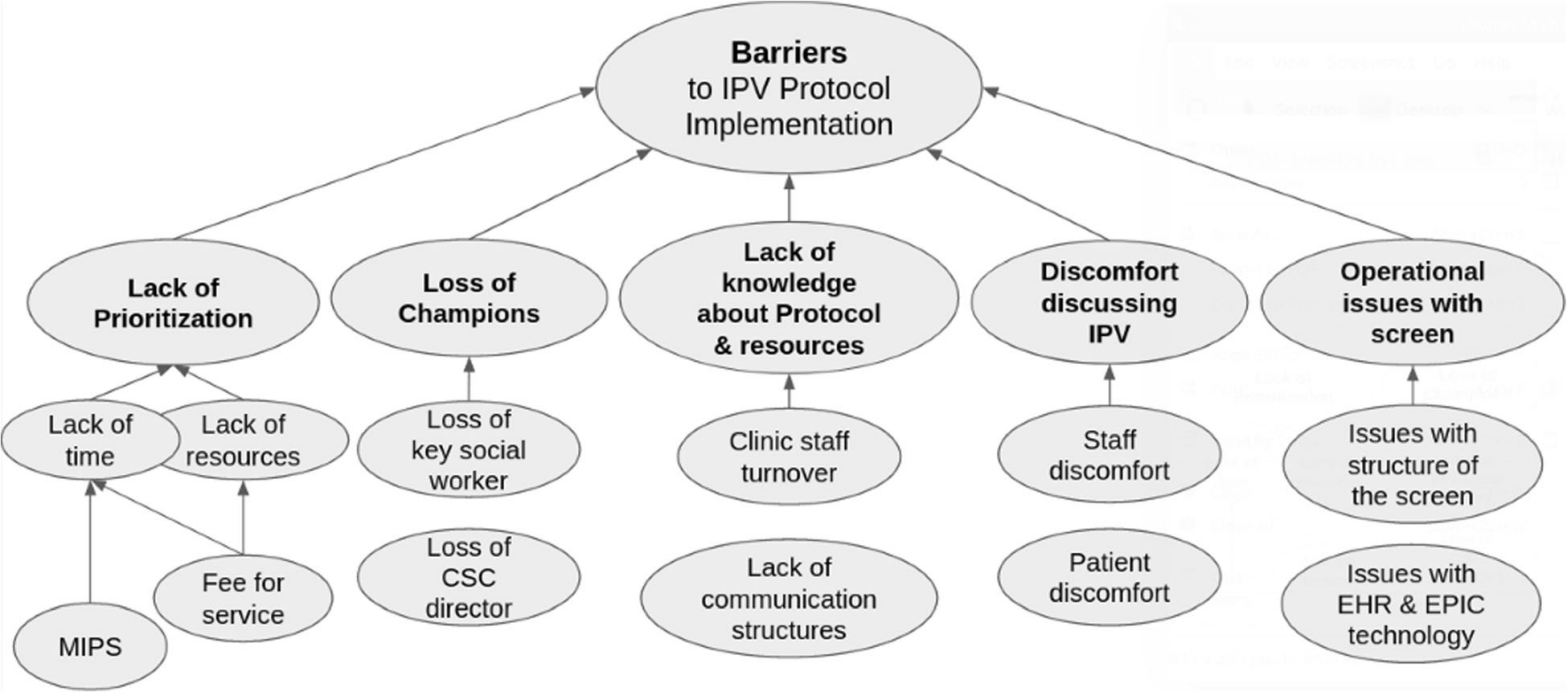
Barriers to IPV Protocol implementation

**Fig. 4:**
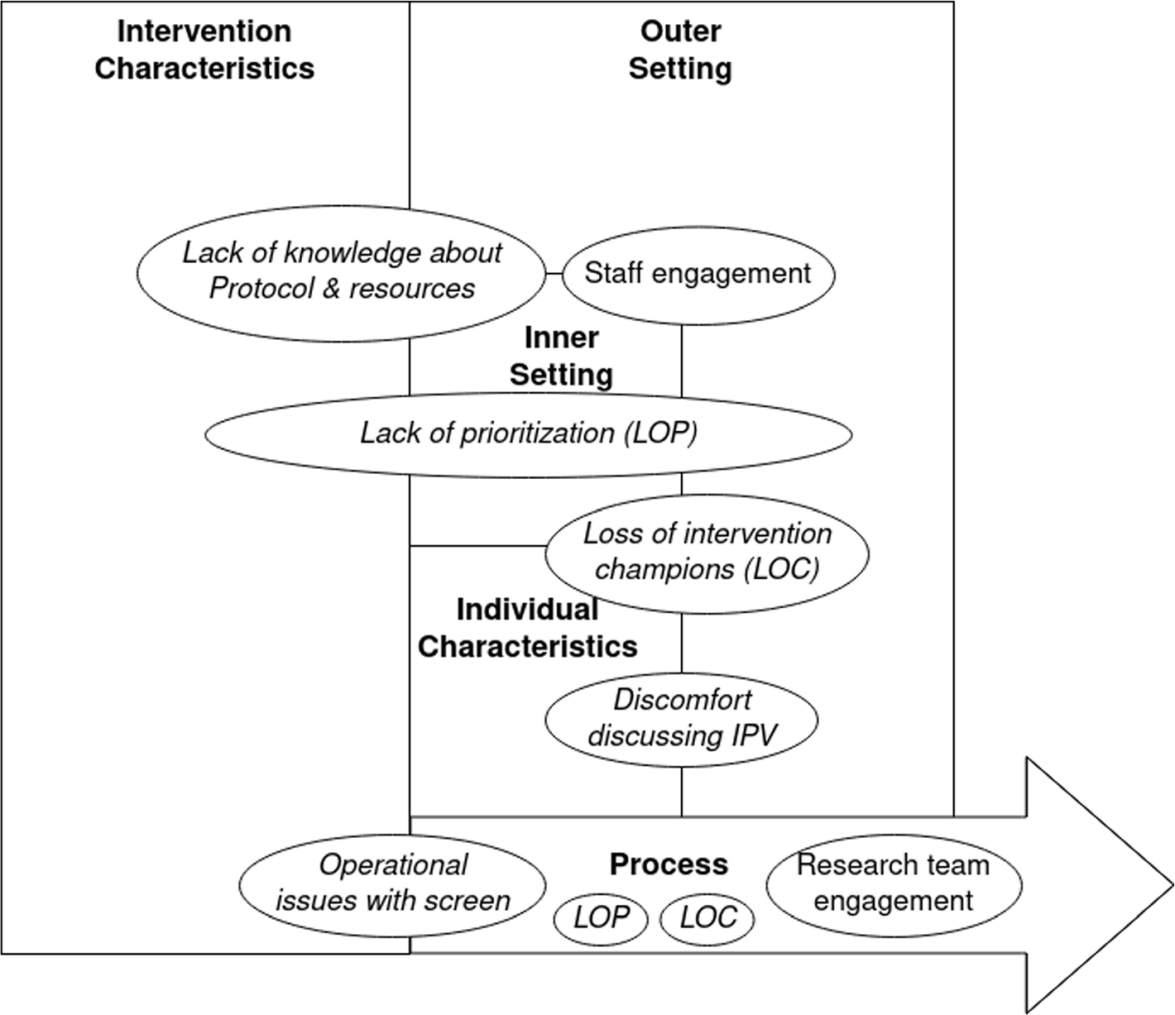
Identified facilitators and barriers to implementation of the M Health Network IPV Protocol mapped to CFIR domains

Key facilitators to intervention implementation were the engagement from clinic managers, rooming staff, and social workers and the engagement and efforts on the part of the research team to design a rigorous, evidence-based IPV protocol, to provide high quality training to clinic staff, and to incorporate feedback from process interviews into the intervention as it progressed (Fig. 2). Barriers to intervention implementation were related to the themes of prioritization; loss of intervention champions (i.e., key stakeholders); lack of knowledge about intervention protocol and resources; staff and patient discomfort discussing IPV; and operational issues with screening (Fig. 3). All identified facilitator and barrier themes repeated across multiple of the five CFIR domains (codes) (Fig. 4). While process interviews were undertaken at three points of time during implementation, temporality or change across time was not always evident in interviews. Most process interviews were done at 12 months, explaining the higher number of quotes included from this time-point.

Despite the barriers to implementation identified, clinic staff believed that when patients screened positive and chose to be referred to care, their care was5 coordinated. Between commencement of the IPV protocol in November 2016 and July 2019, 134,379 CSC patients were screened for IPV, of which 1097 screened positive. Of those who screened positive (56% female), 48% (527) were referred to a CSC social worker or Behavioral Health Team member. Of those referred, 11% (58) were seen either by a CSC social worker, a Behavioral Health Team member, or the caseworker at the community organization. The screening rate for all CSC clinics plus psychiatry in 2017 and 2018 was 32%, and in 2019 it was 16% (Flowers NI, Renner LM, Logeias ME, Wang Q, Morrow G, Clark CJ: A systemic intimate partner violence intervention to identify and support survivors in a multi-specialty health system, in preparation). From the beginning of implementation, there were several patients (including women and men, and both positive and negative screens) who expressed gratitude for the IPV questions being asked.

### Facilitators to implementation

#### Staff engagement

Throughout the phases of the intervention, there was a general understanding on the part of clinic managers, rooming staff, and social workers of the need for standardization of IPV screening and referral in order to provide the best care possible to patients. Clinic managers believed that the IPV screening tool and related trainings helped their clinic staff understand that to provide the best care, a patient needs to be treated holistically, not just for the focus area of the clinic. Specifically, one clinic manager expressed that asking the IPV screening items to all adult patients (not only female-identified) contributed to building awareness of IPV and creating a message that anyone may experience violence. By the end of the intervention, several clinic managers and rooming staff voiced that screening did not take long to do and that it had the potential for great impact.

#### Clinic managers

Clinic managers were aware of the requirement for carrying out the IPV protocol and wanted to support rooming staff in doing it through encouragement and positive reinforcement. Clinic managers were willing to let rooming staff learn and adapt to use of the IPV screening tool and believed it could successfully fit into clinic workflow. However, most recognized that realistically, there was not time to foster deeper learning.

“*No, we try to not have a culture of shame or culture of whatever it is in a negative connotation. We try to make it positive. It's a conversation piece to say, "Hey, guys. We were only 50% compliant or whatever it was. I think we have room for improvement." You get your pompoms out to try that in a positive manner moving forward because obviously, doing the negative, [chuckles] you're not going to get movement on the needle to move it forward.”- Clinic Manager, 12 months*

Although there wasn’t consistent enforcement of the IPV protocol at the facility level, some clinic managers kept track of their staff’s use of all screens and would review this with their teams during “huddles” or on an individual basis if improvement was needed. Some clinics, like primary care, did monthly audits of their team’s use of the screen. Teamwork was important to clinic managers in completing the IPV screen. Many clinic managers believed that implementation was going well and that their staff were able to conduct the screen appropriately.

#### Rooming staff

Rooming staff, in their persistence in performing the IPV screen, were instrumental to this intervention. Rooming staff typically had good rapport with patients, as they interacted with them more consistently than other staff, which facilitated patient comfort in being asked difficult questions. To complement the good rapport, rooming staff also demonstrated flexibility around creating explanatory and/or introductory language to the IPV screening questions. In situations when a patient’s family was in the room, as was often the case in oncology, rooming staff did not complete the IPV screen (the protocol was to ask the questions only if the patient was alone or with a medical interpreter). Inability to do the screen was then documented in EPIC. Rooming staff also demonstrated willingness to talk through why screening was being done with patients who were averse to it and resilience in working through their own discomfort in asking the questions. Rooming staff showed empathy towards patients by trying to imagine what it felt like to be asked questions of such a serious and personal nature. Over time, rooming staff’s comfort in asking the questions increased.

“*There are, when it comes down to the end-user, some of them aren't as comfortable as others. The benefit is that they're learning that it's something that they need to do whether they're comfortable or not and they are learning how to be comfortable. The more they do it the more comfortable that they are. Whereas in the beginning, they were bringing a lot of things to me to address the uncomfortableness and I'm not getting that anymore. I think that it's part of our main language now.”*

*- Clinic Manager, 12 months*

When there was discomfort or cynicism towards the screen, it often took only one positive intervention to win rooming staff over to the importance of the screen.

*“You know, I was very cynical about this when it first started. I thought it was so stupid, but I did it because it was what was expected of me. But the first time I got a positive response where there's a social worker... I said, "Okay, this is a good thing. I don't know that I work in an ENT clinic for ears and it has nothing to do with psychology or primary care. This is one domestic abuse victim who's getting help and wasn't seeming to find it anywhere else."*

*-Rooming Staff, 27 months*

Across clinic settings, rooming staff were very engaged in connecting the patient to care when a positive screen occurred. Yet, there was some frustration expressed by rooming staff when there was a patient who declined being linked to services after screening positive.

“*We've had a handful of positive responses, staff are really upset if when there's a positive response, they refuse help that has happened once or twice”*

*-Clinic Manager, 12 months*

Clinic managers also reported that it was difficult for rooming staff who experienced a positive screen because word of what happened to the patient did not always get back to the clinic.

#### Social workers

Social workers were very receptive to the intervention and appreciative that the CSC was allowing for a more comprehensive look at patients’ psycho-social determinants of health. Social workers facilitated the conversation about community resources and fostered a relationship between clinic staff, social worker, and patient. They made concerted efforts to follow up with patients if they weren’t immediately available.

“*It also opens up a future relationship between myself and them [patient] as well too so that I always invite them to come back with their questions or need check in.”*

*-Social Worker, 12 months*

### Research team engagement

Apart from the engagement of CSC staff members, the research team’s effort to design an evidence-based IPV screening protocol that centered patient choice and utilized the existing structure of the CSC facilitated implementation. The provision of multiple, high quality trainings over the course of implementation provided support to staff members who were tasked with performing the screens to develop competence in asking the questions. The undertaking of process evaluation interviews throughout implementation allowed for opportunities for clinic managers, rooming staff, and social workers to reflect on their use of the IPV protocol, including successes and challenges, and to suggest changes. As implementation progressed, the research team made efforts to adjust the intervention where needed in response to this feedback. The research team also provided periodic screening rate data to the clinic managers and CSC leadership to show progress and encourage adherence throughout implementation.

### Barriers to implementation

#### Prioritization

IPV screening was not a direct financial priority for the CSC, as it fell outside the scope of a traditional fee for service payment model of healthcare.

*"It's not that it's not important but at the end of it all there are certain things I have to do [to bill] and if I don't get to this one, it's not that it's not important, but time is a big factor”*

*-Clinic Manager 12 months.*

Under the fee for service model, social workers were generally considered non-revenue generating. Because of this, they were limited in capacity and resources, impeding a key aspect of the IPV protocol. Due to the low number of social workers and Behavioral Health Team staff, those who worked at the clinic were not always immediately available for care coordination. Even when clinic staff knew how to implement the IPV protocol, there were several reported cases of not being able to reach a social worker when a patient chose to pursue care.

*“We don't have a vast social work amount of people that can just come at the drop of a hat. If we get them alone for that split second and they're willing to talk, it's like we're frantically trying to, "Okay. What are we going to try to do to get this?" and the provider wants to get in the room too.”*

*-Clinic Manager, 12 months*

Fee for service further contributed to time pressure placed on the rooming staff by physicians, a theme that came up repetitively across all phases of the intervention. Rooming staff were expected to room patients as quickly as possible given the volume of care provided, and at times, competing priorities for space. Some rooming staff shared stories of not having private space to talk to a patient who screened positive. Several clinic managers noted inconsistencies when their staff felt that they didn’t have time to do all that was required of them.

*“Sometimes when we become so convinced in our mind that there's not enough time, then we just mentally shut down and we begin taking shortcuts. And that's what I see sometimes on chart audits… when I bring it back up, I hear "well we're in a hurry, were in a hurry".”*

*- Clinic Manager, 12 months*

Occasionally, rooming staff reported they did not have time to check the date of the last IPV screen, which prompted some to repeat the screen every time they saw a patient, which unnecessarily extended the rooming time.

One year into implementation of the intervention, five new and mandatory, merit-based incentive payment system initiatives (MIPS) were undertaken, which contributed to some rooming staff’s lack of time to perform IPV screening. The five new initiatives were connected to financial incentives, and subsequently the IPV screen was not as clearly emphasized given these constraints. The IPV screening tool was included in standardization and learning training for the clinics, but MIPS was the focus. Additionally, physician’s priorities shaped prioritization of the IPV protocol. If the screen was not a physician’s priority, there was often little that a clinic manager could do to prioritize its use.

“*I mean, we, our rooming staff will work based on provider direction. So, if the provider said that this is the number one most important thing then rooming staff would do it. But it's not and that's just the reality ofive new and mandatory, merit-based incentive*

* Clinic.”*

*- Clinic Manager, 12 months*

#### Loss of intervention champions

Staffing changes in intervention champions (i.e., key stakeholders) over the course of the intervention further strained its implementation. The first executive director of the CSC and a Behavioral Health Team social worker were uniquely engaged with early planning and implementation, bolstering organizational support and resources for the intervention. These positions turned over during implementation, and the social worker position was not filled. By mid-implementation, there was detectable confusion about with whom to refer patients to, as the social worker was a key link for IPV care coordination. Some clinic staff felt wary about not being prepared to offer coordinated care to patients who screened positive during this time.

“*But I think we need to know that if we're asking the questions and we're giving the suggestion or the illusion that we have somebody here that can help, we need to make sure that, with [name] being gone, that we do have somebody here to help. Otherwise, why are we asking? We’re giving somebody the idea that we're going to be able to help them and we can’t so we're just as-- Here's the world and there you go. They’ll never ask for help again.” -Rooming Staff, 12 months*

#### Lack of knowledge about intervention protocol and resources

General lack of knowledge about the IPV protocol arose due to a lack of centralized communication channels to reach all clinic staff served as barriers to the IPV protocol routinely functioning as intended. This served as a barrier if a positive screen did occur, especially during the beginning and middle phases of the intervention. The structural chain of care coordination, from rooming staff, to social worker, to community case manager, was not always clear. Some clinic managers and rooming staff did not feel confident that they knew whom to contact or had difficulty reaching social work staff in a timely manner. Some staff were worried about having a patient waiting for resources and not being able to get them connected quickly. Even at the end of the study, some clinic managers worried that in the event of a positive screen, their rooming staff would not know about these resources.

“*Frankly, like I'll just be frank in terms of on the nursing piece, if we did have a positive screen I don't know if they would know about like the card and things like that, that it's just not part of our everyday.”*

*-Clinic Manager, 27 months*

Knowledge of the many resources for implementation (e.g., “success stories” distributed by the research team, the IPV resource cards), the medical interpreters’ awareness of the IPV screening, and the availability of additional trainings for staff, was lacking among both clinic managers and rooming staff, even at the end of implementation. Staff turnover also made it difficult to ensure up-to-date training and dissemination of knowledge to new staff across the course of the intervention.

“*I think what you need to know is that our clinic has had about 100 percent turnover in the last 2 years, so, you're talking to new people all the time, and that's primarily due to our care model change. So, this has been all new training for them and new orientation. Comes back to the whole team.”*

*-Clinic Manager, 27 months*

#### Staff and patient discomfort discussing IPV

There was expression of discomfort in asking the screening questions by some rooming staff throughout implementation. The behaviorally specific nature of the HARK and DA-5 questions contributed to this feeling. Along with this, there was some expression of fear or worry about negative responses from patients. Talking about IPV with patients from varying cultural backgrounds was also mentioned by several rooming staff as a concern due to worry that the screening questions would offend people, especially among cultures they perceived as being male dominant.

“*Or there's a cultural difference. I feel like there are certain cultures that are more – what's the word? Like more male influence. It just makes me feel uncomfortable to ask them if it's violent in their home.”*

*-Rooming Staff, 27 months*

Although numerous trainings were offered, including trainings focusing on how to work with patients from diverse backgrounds, staff across clinics believed more trainings would be beneficial to improve confidence in using the screening tool, learning the warning signs of IPV, and knowing how to talk to patients about IPV.

Patients sometimes refused to answer the IPV screening questions. Patient discomfort in answering questions was reported by some rooming staff from the beginning of implementation and continued throughout. Some patients (often men) reportedly expressed anger or annoyance, sometimes through deflection (nervous laughing or ridiculing), but more often in visible discomfort or awkwardness. Patients were sometimes taken aback by the personal and direct nature of the questions, about not being asked about violence from people other than a partner, or in some cases, because they felt the use of the word “partner” implied a same-sex relationship.

“*I've gotten some really funny answers too like "Listen my wife's half my size she's not going to do any of this to me" or "No because he knows I'd flatten him" or "No, because I would hit him back and call my cousins"*

*-Rooming Staff, 12 months*

Although some patients expressed discomfort in discussing IPV, several rooming staff shared stories of patients thanking them for asking the questions.

“*Oftentimes, it's people who aren't saying yes. It's, "Nope, and I'm really grateful that it's not, but I'm so glad you ask this." There's one patient who always says to me that, "You saved my life." not personally, and she says it every time. Now she answers no to those questions, but she always says, "But I'm so grateful you asked them." -Rooming Staff, 12 months*

#### Operational issues with screening

Finally, issues with the structure of the screening questions and the electronic health record and EPIC technology served as barriers to implementing the intervention. Several rooming staff members expressed that the frequency and repetitiveness of questions sometimes frustrated patients. To compensate for this, some rooming staff reported only asking the general safety question as a precursor to determine whether or not they asked the IPV screen. There was some confusion about whether to continue to ask the IPV screening questions if the first answer to “do you have any concerns about your safety” was a ‘no’. Baseline interviews reflected that some patients’ answers to the Danger Assessment screen were unclear (i.e., not a ‘yes’ or ‘no’) and rooming staff asked if an ‘N/A’ option could be added.

Initially, implementation was planned to be undertaken in thirteen clinics with high female patient populations, with later scale-up to all clinics in the multi-specialty practice. However, given a shared electronic health record and certain common protocols across the organization, it was logistically impossible to limit the scope of the project. Accordingly, it was also difficult to make changes to the screen once it went live and the research team’s control over who had access to the screen was diminished. Throughout implementation, challenges with EPIC were brought up in some of the interviews. Challenges ranged from inability to find the date of last screen or patient’s history to issues with the resources page not being accessible enough.

## Discussion

Over the course of 3 years, the *M Health Network* intervention succeeded in implementing an IPV screening and referral protocol at the CSC, a multi-specialty clinics and surgery center. However, identified barriers may have contributed to impeded ability to consistently screen for IPV every 3 months as the protocol stipulated, the low rates of screening, and the low occurrence of referral. Interviewees believed that when patients screened positive and chose to be linked to care, their care was coordinated, despite contradicting evidence from screening rates and referral data for this project (Flowers NI, Renner LM, Logeias ME, Wang Q, Morrow G, Clark CJ: A systemic intimate partner violence intervention to identify and support survivors in a multi-specialty health system, in preparation). This analysis sheds light on the complexities of implementing an IPV screening and referral protocol in an outpatient, multi-specialty clinical setting, and highlights the viability of using rooming staff to implement the screen. Identified facilitators and barriers corroborate existing evidence and illustrate potential causes of ambivalence towards universal IPV screening in healthcare settings. Findings from this analysis also provide evidence for use of the CFIR framework to guide the process evaluation of a systemic IPV screening and referral intervention in a multi-specialty health system.

The barriers identified in this analysis add to and reaffirm an existing body of literature on barriers to successful IPV screening in the U.S. [20–22] Lack of time, lack of resources, lack of provider knowledge, and discomfort around screening for IPV have all been identified as key barriers to implementing IPV screening in healthcare settings, findings that are reflected in this analysis [20, 21]. In this intervention, lack of time to screen for IPV stemmed from physician’s prioritization of fee for service activities and the introduction of five new and mandated health screens (MIPS) midway through implementation. This indicates that there was a lack of incentive (financial or procedural) to drive IPV screening in this intervention, another documented barrier [35]. Furthermore, these findings contribute to demystifying aspects of physician attitudes toward screening [21, 28, 36, 37]. Lack of resources, specifically lack of available clinic social workers and lack of physical space to talk with patients who screened positive for IPV, made IPV care coordination challenging. While this intervention provided a lot of training and resources to clinic staff, issues with communication and high staff turnover led to people being unaware of the access they had to knowledge and resources. These findings highlight causal factors for lack of provider knowledge for this intervention, and reinforce the importance of both initial and ongoing training [38, 39]. Our findings also add nuance to the evidence that discomfort around discussing IPV is a barrier to IPV screening [20], providing the perspective of the rooming staff implementing the screen and their recounting of their patient’s perspective.

Stakeholder engagement has been identified as key to implementing successful IPV protocols in healthcare settings, corroborated by our finding that the loss of the initial CSC medical director and a key social worker who both championed the intervention presented a barrier to implementation of the IPV protocol [21]. Clinic managers, rooming staff, and social workers were generally supportive of the intervention and worked diligently to fit the protocol into their existing workflows, as well as to overcome the barriers to implementation that arose. Rooming staff were flexible and creative in making the screening work, showed resilience in working through their own discomfort in asking the questions, showed care for their patients, showed a dedication to meeting expectations placed on them, and showed a desire to learn more about the issue of IPV. Clinic managers were engaged in leading their staff to implement the screen, recognized the importance of it, and advocated for their staff. Social workers worked diligently to fulfill their role in care coordination.

The research team played a key role in ensuring this IPV protocol was implemented, through proposing the idea, designing the protocol, training clinic staff in implementation of the protocol, and checking in throughout implementation to gather process data. The research team’s efforts were facilitated by support from the first CSC executive director and a key social worker. The IPV protocol was ongoing as of 2019 at the CSC, but the rate of screening dropped from 32% in 2017 and 2018, to 16% in 2019 after the research team withdrew following intervention completion (Flowers NI, Renner LM, Logeias ME, Wang Q, Morrow G, Clark CJ: A systemic intimate partner violence intervention to identify and support survivors in a multi-specialty health system, in preparation). It is unclear whether an intervention like this could have been implemented at the CSC without the concerted efforts of the research team (or another group dedicated to implementation) and the dedicated efforts of clinic managers, rooming staff, and social workers.

Importantly, these findings reveal several concrete strategies for troubleshooting common barriers to implementing an IPV screening and referral protocol in a clinical setting:

- *Engage key stakeholders continuously to foster ongoing support and prioritization of the intervention and to address any loss of intervention champions.*
- *Set up communication structures within clinics and the broader medical setting prior to commencing implementation to address lack of knowledge about the IPV protocol and the resources available for clinics to prepare their staff.*
- *Mandate physician training on the IPV protocol, even if they are not involved in implementation of screening or referral, to address lack of physician prioritization.*
- *Provide mandated refresher training for the staff implementing the IPV protocol at frequent time-intervals throughout implementation to address high staff turnover and subsequent lack of knowledge about the IPV protocol.*

Situational barriers to implementing the IPV protocol were difficult to avoid or address, such as the introduction of five new mandated screens (MIPS), and technological issues with the electronic health record and EPIC screening tool. These barriers were overcome due to effort from the research team and clinic staff. Other barriers, such as fee for service and discomfort around talking about IPV, were structural in nature and difficult to avoid or address. Fee for service, a commonly used payment model in U.S. healthcare, contributed to lack of time and lack of resources, which drove lack of prioritization of the IPV protocol. Discomfort when talking about IPV is unavoidable. The only way to address this major, common barrier is to work diligently to support survivors of IPV and to destigmatize victimization.

### Strengths and weaknesses

This analysis uniquely adds to the body of literature on IPV screening in healthcare settings in the United States in several ways. It investigates the facilitators and barriers to implementing a unique IPV screening intervention (i.e., all patients screened regardless of gender, rooming staff implement the screen) at a large, multi-specialty clinic and surgery center. Implementation of the intervention was guided by the CFIR framework, as was the gathering of process data throughout the 3 years of implementation, and the analyzing of the process data post-implementation. Individual and group process interviews with clinic managers, rooming staff, social workers, and the CSC executive director conducted across different phases of the intervention provided rich qualitative data to assess implementation facilitators and barriers. Process data were bolstered by inclusion of data from post-implementation semi-structured, informal interviews with the research team members.

While the CFIR framework is evidence-based and thorough, such that content assessed did not fall outside of the 5 core domains and numerous constructs, using it added to the complexity of analyzing the data for this analysis. Complexity was due in large part to interrelated content being assessed across many of the code sub-domains which led to difficulty in achieving inter-rater reliability of the five domains and associated constructs, even with the written definitions from the website readily available. This challenge has been reported by other applications of the CFIR [40]. To compensate, coders first coded data using the five broad CFIR domains, then re-coded data coded into the five domains using the associated constructs. The emergent themes presented in the results often overlapped across the five CFIR domains. Another issue was that the domains and constructs of the CFIR do not clearly reflect change over time. Thus, while this analysis had data from three implementation time-points, temporality was not clear in the findings. Overall, the application of the CFIR was critical to being able to identify barriers and facilitators of implementation, and the nuance in the sub-constructs is particularly useful for intervention planning. However, a focused data coding, wherein the text segments would be coded with the most salient domain and sub-construct instead of all of the nuanced and potentially overlapping domains, as have been employed in prior CFIR research would have greatly reduced the coded data for analysis and reduced overlap across domains [30].

Process interviews captured data on the screening aspect of this intervention, but did not fully capture referral. While there was one interview with a clinic social worker involved in care coordination, it focused on the perspectives of referral that related to communication with clinic managers and rooming staff. The component of the IPV protocol that involved referring patients to case management at the community organization was not captured in the interviews. This may have been due to an effort to ensure patient privacy. Due to lack of data, it is impossible to know if the low referral (48% of patients who screened positive were referred to care) was attributable to the barriers to implementation identified in this analysis or to patients declining referral (Flowers NI, Renner LM, Logeias ME, Wang Q, Morrow G, Clark CJ: A systemic intimate partner violence intervention to identify and support survivors in a multi-specialty health system, in preparation). Further process review of the CSC – community service provider partnership and the process of referral would provide additional, critical insights into sustainable systemic IPV screening and referral protocols given the importance of partnerships to address this inherently multidisciplinary, social determinant of health.

The *M Health Network* IPV screening and referral protocol was implemented but faced common barriers to implementation over the course of 3 years. The efforts of the research team, clinic managers, rooming staff, and social workers allowed most barriers to be overcome. Rooming staff are highly capable of implementing IPV screening and uniquely positioned to do so due to their proximal relationships with patients. CFIR is a complex, but comprehensive, tool to guide process evaluation for public health interventions in multi-specialty health systems in the U.S.

## Supplementary information

**Additional file 1.** Process Evaluation Questions.

## Data Availability

Data sharing is not applicable to this article as no data sets were generated or analyzed during the current study. The *Template for Intervention Description and Replication* checklist was used and is attached as an additional file.

## Abbreviations

IPV: Intimate Partner Violence
USPSTF: U.S. Preventive Services Task Force
CFIR: Consolidated Framework for Implementation Research
M Health: University of Minnesota Health
M Health Network: *The Coordinated Care for IPV Survivors through the M Health Community Network*
CSC: Clinic and Surgery Center
HARK: Humiliation, Afraid, Rape, Kick
DA-5: Danger Assessment
MIPS: Merit-based incentive payment system
NAM: National Academy of Medicine
