# Supplementary material for: Process evaluation of a systemic intervention to identify and support partner violence survivors in a multi-specialty health system
Source: BMC Health Serv Res. 2020 Oct 31;20:996. doi: 10.1186/s12913-020-05809-y (PMC7603748; doi:10.1186/s12913-020-05809-y)
Supplement: Supplementary file 1 — Additional file 1. Process Evaluation Questions. [file 12913_2020_5809_MOESM1_ESM.pdf]

Process Evaluation Questions

Coordinated Care for IPV Survivors through the M Health Community Network

Process evaluation questions – 3 Months Post

1. The Partner Violence screen was added to EPIC in mid-November 2016.
   - To what extent are staff using the screen?
   - Is the screen being used with all adult patients?

2. What is going well with respect to the use of the Partner Violence screen?
   - What have been any positive changes to practice
     - Positive changed in staff-patient interactions?
     - More comfort with responding to disclosures to IPV?
     - More comfort knowing next steps for IPV-positive patients?
     - Changing climate and supports around clinic? Increases in discussions of IPV among staff?
   - Any success stories to share?
     - Anything that went particularly well in the process or any patient you encountered where you feel this new screen and response process made a positive difference?

3. What challenges have there been encountered with respect to the use of the Partner Violence screen?
   - Challenges in using screen?
     - Completing in time allowed for rooming process; getting patient alone; asking the questions; documenting responses in the electronic medical record; knowing when or when not to complete screen; connecting with clinic social workers?
   - What have you done to problem-solve these challenges?
     - What has worked?

4. What training or supports do your staff need around the topic of IPV?

Midpoint process evaluation questions for Administrator

1) Assess Administrator’s knowledge about the IPV grant

2) Ask:

- What policies, regulations, and guidelines influence how this clinic system identifies and responds to patients who experience IPV? (Outer: External policies)

- What is the priority of screening and responding to IPV relative to other initiatives that are happening now in this clinic system? (Inner: relative priority)

- How does implementation of the revised IPV screening and response protocol align with the organizational goals in the clinic system? (Inner: goals and feedback)

- How well does the revised IPV screening and response protocol fit with existing work processes and practices in the clinic system? (Inner: compatibility)

- What are some of the organizational barriers to meeting the needs of patients who experience IPV? (Outer: patient needs and resources)

- How does the size of the clinic system and diversity of clinics affect how the IPV screen and response protocol is carried out? (Inner: Structural characteristics)

- What are your plans for sustainability of the IPV screen and response intervention after the initial grant ends in 2018? (Inner: leadership engagement)

3) Assess Administrator’s willingness to support the project from an Administrative perspective, interest to participate in monthly team meetings, etc.

Midpoint process evaluation questions for Clinic Managers

1. Since the IPV screen and response was revised in November 2016, what has been the general level of receptivity in your clinic to implementing the revised IPV screening and response protocol? (Inner: Implementation climate)

   a) To what extent are rooming staff in your clinic implementing the IPV screen and response protocol according to its design (e.g., all adults, every three months, documented referral options) (Process: execute)

   b) How well does the revised IPV screening and response protocol fit with existing work processes and practices in your clinic? (Inner: compatibility)

   c) How well do you think IPV screening and response protocol meets the needs of the patients served in your clinic? (Outer: patient needs and resources)

d) How do staff in your clinic problem-solve issues around implementation of the IPV screen and response protocol? (Process)

2. What is the priority of screening and responding to IPV relative to other initiatives that are happening now in your clinic? (Inner: relative priority)

3. What are the benefits to complying with the protocol? (Inner: Implementation climate)

4. What are the sanctions for not complying? (Inner: Implementation climate)

5. Since the IPV screen and response was revised in November 2016, how has conversation around IPV and patient care in your clinic changed? (Inner: communication)

6. What endorsement or support have you seen or heard from leadership with respect to the IPV screen and response intervention? (Inner: leadership engagement)

7. What kind of support or actions do you need from leadership to help make implementation of the revised IPV screening and response protocol more successful? (Inner: leadership engagement)

8. Who in your clinic has been championing the IPV screening and response protocol and what type of behaviors have they been exhibiting through this process? (Process: engage)

9. What resources would you like to receive to help make implementation of the revised IPV screening and response protocol more successful? (Inner: available resources)

Midpoint process evaluation questions for Rooming Staff

1. Since the IPV screen and response was revised in November 2016, what has been the general level of receptivity in your clinic to implementing the revised IPV screening and response protocol? (Inner: Implementation climate)

    a. To what extent are you (is your clinic) implementing the IPV screen and response protocol according to its design (e.g., all adults, every three months, documented referral options) (Process: execute)

    b. How well does the revised IPV screening and response protocol fit with existing work processes and practices in your clinic? (Inner: compatibility)

2. What positive interactions with patients have resulted from implementation of the IPV screen and response protocol?

3. What challenges do you have with implementation of the IPV screen and response protocol and how do staff in your clinic problem-solve issues around implementation?

4. How well do you think IPV screening and response protocol meets the needs of the patients served in your clinic? (Outer: patient needs and resources)

    a. How could the screening and response protocol be improved to better meet patient needs?

5. Since the IPV screen and response was revised in November 2016, how has conversation around IPV and patient care in your clinic changed? (Inner: communication)

6. What is the priority of screening and responding to IPV relative to other initiatives that are happening now in your clinic? (Inner: relative priority)

7. Who do you ask if you have questions about the intervention or its implementation? (Inner: access to knowledge)

8. What kind of support or actions do you need from leadership to help make implementation of the revised IPV screening and response protocol more successful? (Inner: leadership engagement)

9. What resources would you like to receive to help make implementation of the revised IPV screening and response protocol more successful? (Inner: available resources)

Endpoint process evaluation questions for Clinic Managers

1. Since the IPV screen and response was revised in November 2016, how has the general level of receptivity to implementing the revised IPV screening and response protocol changed over the past two years? (Inner: Implementation climate)

    a) To what extent are rooming staff in your clinic implementing the IPV screen and response protocol according to its design (e.g., all adults, every three months, documented referral options) (Process: execute)

    b) How well does the revised IPV screening and response protocol fit with existing work processes and practices in your clinic? (Inner: compatibility)

    c) How do staff in your clinic problem-solve issues around implementation of the IPV screen and response protocol? (Process)

2. What is the priority of screening and responding to IPV relative to other initiatives that are happening now in your clinic? (Inner: relative priority)

3. What are the benefits to complying with the protocol? What are the sanctions for not complying? (Inner: Implementation climate)

4. What endorsement or support have you seen or heard from leadership with respect to the IPV screen and response intervention? (Inner: leadership engagement) What negative response have you seen or heard from leadership or other clinic managers with respect to the IPV screen and response intervention?

5. Since the IPV screen and response was revised in November 2016, how has conversation around IPV and patient care in your clinic changed? (Inner: communication)

6. How well do you think IPV screening and response protocol meets the needs of the patients served in your clinic? (Outer: patient needs and resources)

7. In your opinion, should your clinic continue to screen adult patients for IPV? If so, would you suggest any changes to the screen or response protocol?

Endpoint process evaluation questions for Rooming Staff

1. Since the IPV screen and response was revised in November 2016, how has the general level of receptivity to implementing the revised IPV screening and response protocol changed over the past two years? (Inner: Implementation climate)

    a. To what extent are you (rooming staff in your clinic) implementing the IPV screen and response protocol according to its design (e.g., all adults, every three months, documented referral options) (Process: execute)

    b. How well does the revised IPV screening and response protocol fit with existing work processes and practices in your clinic? (Inner: compatibility)

2. How well do you think IPV screening and response protocol meets the needs of the patients served in your clinic? (Outer: patient needs and resources)

    a. What challenges do you have with implementation of the IPV screen and response protocol? How do you problem-solve issues around implementation?

    b. How could the screening and response protocol be improved to better meet patient needs?

    c. What positive interactions with patients have resulted from implementation of the IPV screen and response protocol?

3. Since the IPV screen and response was revised in November 2016, how has conversation around IPV and patient care in your clinic changed? (Inner: communication)

4. What endorsement or support have you seen or heard from leadership with respect to

the IPV screen and response intervention? (Inner: leadership engagement) What negative response have you seen or heard from leadership, your or other clinic managers with respect to the IPV screen and response intervention?

5. What is the priority of screening and responding to IPV relative to other initiatives that are happening now in your clinic? (Inner: relative priority)

6. In your opinion, should your clinic continue to screen adult patients for IPV?

7. If so, would you suggest any changes to the screen or response protocol?
